# A Methodological Proposal for Health Technology Assessments: A Case Study on Biosimilar Drugs

**DOI:** 10.3390/jmahp13020012

**Published:** 2025-03-31

**Authors:** Marilisa Pia Dimmito, Lisa Marinelli, Eleonora Chiara Toto, Giuseppe Di Biase, Ivana Cacciatore, Pierpaolo Toto, Michele Ciulla, Benedetta Monti, Fiorenzo Santoleri, Alberto Costantini, Antonio Di Stefano

**Affiliations:** 1 Department of Pharmacy, University “G. d’Annunzio” of Chieti-Pescara, Via dei Vestini 31, 66100 Chieti, Italy; marilisa.dimmito@unich.it (M.P.D.); eleonora.toto@unich.it (E.C.T.); giuseppe.dibiase@unich.it (G.D.B.); ivana.cacciatore@unich.it (I.C.); pierpaolototo@gmail.com (P.T.); michele.ciulla@unich.it (M.C.); antonio.distefano@unich.it (A.D.S.); 2 ASL Benevento U.O.C. Assistenza Farmaceutica, Via Trieste e Trento, 82100 Benevento, Italy; b.monti@studenti.unisa.it; 3 Pharmacy of Spirito Santo Hospital, Pescara 65124, Italy; fiorenzosantoleri@hotmail.com (F.S.); alberto.costantini@asl.pe.it (A.C.)

**Keywords:** biological drugs, biosimilars, economic evaluation, healthcare, health technology assessment, pharmaceutical spending

## Abstract

This work proposes a methodological approach that could be useful in multidisciplinary health technology assessments (HTAs). Mathematical models based on real data were used to make predictions for the initial price and actual cost of three classes of biological drugs. Through a comparison of real data, with the data derived through this approach, degree coefficients were formulated to rank the negotiating capabilities of Italian regions. The proposed method could represent a valid means of support for healthcare decisionmakers in planning and reducing pharmaceutical spending, evaluating data, and finding uses for particular medical technologies. This study could be a useful tool for achieving the objectives of HTAs, providing a means of analysis that can be adapted to any data, which may be useful for rationalizing the use of health technologies, reducing waste, and optimally reallocating resources.

## 1. Introduction

Health technology assessments (HTAs) are a multidisciplinary approach used to analyze the medical/clinical, social, organizational, economic, ethical, and legal implications of a particular technology through an evaluation of different parameters, such as its effectiveness, safety, cost, and social and organizational impacts [1,2,3]. Their main objective is to assess the real and potential influences of a technology; the effects that implementing or omitting its use may have on the healthcare system, the economy, and society; and other significant outcomes [4,5,6]. To achieve the shared goal of ensuring that healthcare services are efficient and limiting the constant increases in pharmaceutical spending, greater attention to the resource allocation of health technologies is required to determine an adequate cost–benefit ratio. The Italian healthcare system, known as the Sistema Sanitario Nazionale (SSN), is designed to provide universal access to healthcare, ensuring that everyone can receive necessary care. It is a public system, primarily funded through taxes. Most healthcare services are provided free of charge or have a minimal cost, although the level of funding and services can vary by region, as healthcare is managed at the regional level. The SSN covers a wide range of medical services, including hospital care, specialist visits, and emergency care. For certain treatments, patients may need to contribute; this depends on the region and the type of service. In a context where each region must support its own health system, which represents a significant portion of the budget, it is essential to apply principles and methodologies that allow for the maximization of used resources, achieving effective results at sustainable costs [7]. Using HTA tools, it is possible to establish and share the criteria used in organizational and management decisions, such as when choosing whether to invest in advantageous and innovative technologies or even whether to disinvest in outdated and inappropriate medical services and technologies [8,9]. The patient benefits from the results proposed by HTAs, as they provide objective comparisons that indicate the best treatment for a particular pathology. In this context, economic evaluations play a crucial role in supporting decisions; however, they also have a cost as they require qualified personnel [10]. For this reason, it is advisable to carry out a preliminary analysis to understand whether an evaluation is necessary or not. In this work, mono-criterial HTA techniques are explored in depth. These techniques, including cost–benefit, cost-effectiveness, cost–utility, and cost-minimization analyses, and have the advantage of being easy to apply because each element of the problem is well-defined and the objective is always explicit and quantifiable [11,12]. Unfortunately, an analysis of mono-criteria has the disadvantage of not adequately representing complex contexts [13]. This work aims to carry out a socioeconomic analysis of the utilization of biosimilar drugs, which represent an alternative therapeutic option to traditional biological drugs [14]. For this purpose, an economic analysis was performed, and mathematical models were proposed based on real data. These allowed us to obtain predictions of both the prices and costs of three classes of biological drugs. Through a comparison of real data with those derived from the functions, degree coefficients were formulated to rank the negotiating capabilities of the Italian regions. The proposed approach could represent a valid means of support for healthcare decisionmakers when planning, reducing pharmaceutical spending, evaluating data, finding new strategies for a particular medical technology, or strengthening their purchasing ability. This study could be a useful tool for achieving the objectives of HTAs, rationalizing the use of health technologies, reducing waste, and optimally reallocating resources.

## 2. Materials and Methods

### Economic Analysis

The data used in this work were sourced from the Italian Medicines Agency (AIFA), specifically the “OsMed Report: The Use of Drugs in Italy” released by the AIFA [15], and the “Report on Biosimilars” [16], annually released by this regulatory agency. The 2017 report edition was selected due to its inclusion of sub-reports, which provided separate data for each Italian region, instead of the usual aggregate data. To facilitate the cost-minimization analysis, the purchased drugs, measured in terms of the number of packages, were incorporated into a crucial dataset. Although this specific information was not explicitly provided in the AIFA reports, it was extrapolated from the “expenditure data per capita”. This approach aligns with the methodology recommended by the Ministry of Health in the “Guidelines for monitoring spending and consumption pharmaceuticals” [17]. As prescribed in the guidelines, the per capita expenditure was calculated using the following Formula (1):

(1) $$Expenditure\;data\;per\;capita=\frac{\Sigma {(q}_{i}\times p_{i})}{assisted\;population}$$

where *i*, *q*, and *p*, represent the ministerial code, the number of packages, and the price per package, respectively. In the calculation of the per capita expenditure for each drug, the summation (Σ) is eliminated. The report provides both per capita spending data and the prices charged to individual regions. Furthermore, for the “assisted population”, the number of inhabitants in the Italian regions in 2017 was considered, sourced from the National Institute of Statistics (ISTAT) database [18]. The obtained data were processed and plotted to study the trends of biosimilar utilization rates across Italian regions, as a function of the considered variables. Initially, with the costs kept constant for a package purchased by the Abruzzo region, the economic savings were examined as a function of the proportion of biosimilars used, considering a scale ranging from 0 to 100%.

Based on the law of supply and demand, it was hypothesized that by increasing the utilization of biosimilars, their price would decrease. Starting from this consideration, a function to obtain an “adaptive” average price was proposed. This function considers the following: (a) the growing use of biosimilars, which have a lower price than the originator; (b) the reduction in the unit price due to the increase in the purchase quantities of the biosimilar, in agreement with the common pricing mechanism related to the quantity discount. Subsequently, as it is reasonable that with increasing biosimilar utilization, demand for the originator is reduced, with a subsequent escalation in cost, a function capable of defying the aggregate pharmaceutical expenditure was formulated, which integrates equations representing both the biosimilar (2) and the originator (3), as follows:

(2) t=cx+d

(3) l=mx+n

where *t* and *l* represent the biosimilar and originator unit prices, respectively, *c* represents the slope, *x* represents the utilization percentage, and d represents the intercept at the origin of the line. These functions account for both the biosimilar’s contribution (fraction *tx*) and the originator’s contribution (fraction *l*(1 − *x*)). By combining Equations (2) and (3), we can derive Equation (4).

(4) $$\begin{array}{l} y=l\left(1-x\right)+tx\\ =x^{2}\left(-m+c\right)+x\left(m-n+d\right)+n \end{array}$$

Ultimately, the total cost of pharmaceutical spending, based on the hypotheses made, can be expressed as the following parabolic equation:

$$y=\left(c-m\right)x^{2}+\left(m+d-n\right)x+n$$

Based on the obtained results, to better align the trend of the function with the real data, analogous dispersion line equations were used by replacing the percentages of utilization with their respective quantities (measured in terms of the number of packages). This involved introducing *q_b,_ q_o_*, and *q_T_*, representing the biosimilar, originator, and their total amounts, respectively. Using these quantities, Equations (5) and (6) can be rewritten as follows:

(5) $$t=cq_{b}+d\;with\;q_{b}=q_{T}x\;$$

(6) $$l=mq_{o}+n\;with\;q_{o}=q_{T}\left(1-x\right)$$

where the total cost of pharmaceutical spending, in terms of quantity, is given by the following function:

(7) $$S_{tot}=tq_{b}+lq_{o}$$

to obtain the following Equation (8):

(8) $$S_{tot}=\left(cq_{T}x+d\right)q_{b}+[mq_{T}\left(1-x\right)+n]q_{o}$$

$$\;\;\;\;\;\;\;\;\;\;\;\;\;\;\;\;\;\;\;\;\;\;\;\;\;\;\;=cq_{T}^{2}x^{2}+dq_{T}x+mq_{T}^{2}-2mq_{T}^{2}x+nq_{T}+mq_{T}^{2}x^{2}-nq_{T}x$$

Therefore, considering the hypotheses made, the total cost of pharmaceutical spending, in terms of quantity, is represented by the following parabolic function:

$$S_{tot}=x^{2}\left(cq_{T}^{2}+mq_{T}^{2}\right)+x\left(dq_{T}-2mq_{T}^{2}-nq_{T}\right)+mq_{T}^{2}+nq_{T}$$

Equation (8) does not derive an average price value; rather it determines the total expenditure as a function of the specific quantity. The average price, expressed by *p*, was determined based on the number of packages, obtained using Equations (9) and (10).

(9) $$p=\frac{x^{2}\left(cq_{T}^{2}+mq_{T}^{2}\right)+x\left(dq_{T}-2mq_{T}^{2}-nq_{T}\right)+mq_{T}^{2}+nq_{T}}{q_{T}}$$

(10) $$p=\frac{x^{2}\left(cq_{T}^{2}+mq_{T}^{2}\right)+x\left(dq_{T}-2mq_{T}^{2}-nq_{T}\right)+mq_{T}^{2}+nq_{T}}{q_{T}}$$

Function (10) enabled the formulation of hypotheses concerning the trajectory of drug pricing across different criteria, thereby determining the actual expenditure patterns observed in different regions of Italy through a mathematical construct. This function accommodates variations in spending between regions with a high utilization rate of biosimilars and those with a lower utilization rate. Additionally, it facilitates an examination of pharmaceutical expenditure in regions capable of negotiating purchase prices effectively. The purpose of quantifying deviations from the theoretical value represented by the function is to determine Index (11), which allows for the ranking of regions based on their negotiating ability, as follows:

(11) $$K_{pt}=\frac{P-P_{t}}{P_{t}}$$

where *P* and *P_t_* represent the real and the theoretical prices, respectively.

Negative values of Index (11) indicate regions that pay less for a drug than the ideal price trend, meaning they have a high negotiating capacity. On the other hand, a low negotiation capacity is described by positive values, in cases of regions that pay more for a drug than the ideal price trend.

## 3. Results and Discussion

### 3.1. Experimental Design

The 2017 OsMed report provides information on biosimilars for the following classes of biological drugs: epoetin, growth factors, somatropin, insulin glargine, follitropin, infliximab, etanercept, and rituximab. The selected drugs are those whose originators have lost their patent protection and whose spending impact is among the highest in Italy. Based on an examination and comparison of the data related to these classes of drugs, we decided to apply a cost minimization analysis and a subsequent evaluation limiting the investigation to epoetin, etanercept, and follitropin. A key limitation of this study is the lack of validation against other HTA studies, which diminishes the comparative reliability of the proposed analysis. The reliance on data only from the AIFA 2017 report further restricts the generalizability of the findings. However, the primary aim of this work is to present a general model that can be applied to similar contexts, rather than to provide definitive, region-specific conclusions. While comparing the results with other HTA results in Europe could boost the methodology, the focus here is on developing a flexible framework that can be adapted to various pharmaceutical contexts. The data used in the economic analysis, regarding the three classes of drugs, are reported in Table 1, Table 2 and Table 3. These tables summarize national information as well as individual data from the nineteen Italian regions and the two autonomous provinces of Trento and Bolzano. Some data were directly reported as published in the AIFA reports, while others, such as quantities, were indirectly extrapolated. Regardless of the prices, which, in Italy, can vary from region to region, the aim of the work is to offer an analysis method. Therefore, the quantification of the prices can be considered indicative. Data excluded from the economic evaluation are highlighted in bold. The national data were excluded in all three cases, as they already represent aggregated data and, therefore, are redundant. The regions are ordered in the tables based on decreasing percentages of biosimilar utilization, except for regions excluded from the evaluation, which are listed at the bottom regardless of their utilization percentages. Specifically, Lombardy was omitted from this investigation due to the absence of its biosimilar pricing data in the AIFA tables. Moreover, Aosta Valley and Molise were not considered due to their irrelevant quantities of biosimilars (5 and 36 units, respectively), rendering them statistically insignificant. As shown in Table 1, with an amount of nearly 6 million packages, epoetins are biological drugs that exhibit high variability, ranging from 10.4% to 95.4% in Calabria and Emilia Romagna, respectively. These percentages seem to be distributed on a geographical basis; except for a few cases, the southern regions correspond to lower percentages of biosimilars utilized compared to the northern regions. Abruzzo, ranking 15th, demonstrates intermediate utilization (50.2%). The data for Aosta Valley were discarded, as the AIFA data sheets showed the same prices for the biosimilars and the originators, making their verification difficult.

Furthermore, the value of EUR 5.26 is implausible when compared with the prices observed in other regions, including the much more populous ones (i.e., the Aosta Valley). The region with the best price was Piedmont, with a value more than double (EUR 11.49) that mentioned above. In Table 2, it is evident that etanercept also exhibits considerable geographic variability, ranging from 8.9% in Molise to 97.6% in Tuscany, with a trend of increasing usage from the southern to the northern regions. Notably, in Abruzzo, utilization stands at a lower rate of 13.8%. The data for Sardinia were excluded due to challenges in verifying the pricing, where the biosimilar price exceeded that of the originator.

Follitropin appears to be less extensively utilized, with only a few hundred packages purchased in some regions (Table 3). Interestingly, there is a notable deviation in the trend concerning the biosimilar’s performance compared to the two previous drugs. The utilization percentages remained low across Italian regions, ranging between 4% and 30%, except for Abruzzo, which was the leading region, with a utilization rate of 55.4%. Additionally, the geographical distribution does not reflect the trend observed with previous drugs, with northern and southern regions spread throughout the list. Similar to those of epoetin, the data on follitropin for Lombardy were excluded due to the absence of the prices of the biosimilars in the AIFA tables. The data for Aosta Valley and Molise were discarded, as the low quantities of the biosimilar (5 and 36 units) were not considered statistically significant. The limited use of follitropin biosimilars are probably derived from their recent marketing, since the two biosimilars bemfola and ovaleap were marketed in 2015 and 2016, respectively, only two years before the data collection. Considering the use of epoetin, this hypothesis is valid. The utilization rates of the biosimilars binocrit and retacrit, whose marketing is less recent and dates to 2008 and 2012, were close to 100% in some Italian regions. On the contrary, the same criterion does not apply to etanercept, whose biosimilar benepali is widely used in some regions despite its marketing only dating back to 2016.

### 3.2. Linear Projection of Expenditure

**Figure 1:**
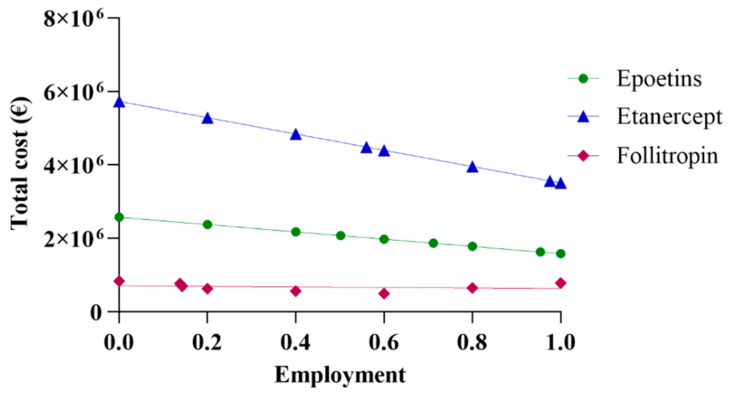
Linear projection of expenditure for epoetin, etanercept, and follitropin.

The initial assessment included a linear extrapolation of expenditure for the three drugs based on data acquired from Abruzzo. The total cost was derived by aggregating the data on expenditure regarding both biosimilars and originators. By progressively incorporating higher percentages of biosimilars, and, thus, decreasing the percentages of originators, a linear projection trend of expenditure was generated. Figure 1 shows the real spending values in Abruzzo, alongside projections corresponding to the region’s utilization rate using the same percentage as the national average and the rate of most efficient region. Based on the data reported in Figure 1, it can be observed that pharmaceutical spending in Abruzzo for epoetin was EUR 2,075,928, a cost derived from the utilization of 64,032 packages. This expenditure for epoetin consisted of 50.2% for biosimilars and 49.8% for the originator, priced at EUR 24.71 and EUR 40.19 per unit, respectively. The projected total expenditure varies between EUR 2,573,582 and EUR 1,582,239, with a percentage difference of −38.52% between the upper and lower limits. An interesting scenario emerges when further exploring Abruzzo’s pharmaceutical expenditure. Specifically, applying the same percentage as the Italian average yields a projected expenditure of EUR 1,867,746, reflecting a decrease of −10.3%. Meanwhile, applying the same percentage as the most efficient region, Emilia Romagna, results in a projected expenditure of EUR 1,627,840, representing a decrease of −21.58%. In a hypothetical situation where only biosimilar epoetins are utilized, the projected expenditure would be EUR 1,582,239, reflecting a decrease of −23.78%.

For etanercept, the pharmaceutical expenditure in Abruzzo was EUR 5,421,213, with the utilization of 6663 packages. This expenditure breakdown includes 13.8% for biosimilar usage, priced at EUR 527.03 per package, and 86.2% for the originator, priced at EUR 859.56 per package. When projecting the total expenditure, it ranges from EUR 5,726,956 to EUR 3,511,426, indicating a percentage difference of −38.69% between the highest and lowest estimates. Further analysis reveals a scenario in which applying the same percentage as the Italian average would result in a predicted expenditure of EUR 4,484,043, corresponding to a decrease of −17.29%. On the contrary, considering the same percentage as the most efficient region, Tuscany, yields a projected expenditure of EUR 3,564,598, representing a decrease of −34.25%. In a hypothetical scenario where only biosimilar etanercepts are utilized, the projected expenditure would be EUR 3,511,426, reflecting a decrease of −35.23%. Regarding follitropin, Abruzzo’s pharmaceutical expenditure amounted to EUR 647,901, considering the utilization of 4278 packages. This expenditure comprised 55.4% for biosimilar usage, priced at EUR 116.47 per package, and 44.6% for the originator variant, priced at EUR 195.00 per package. The total expenditure projection ranges from EUR 834,272 to EUR 497,862, with a percentage delta of −40.32% between the extremes. Abruzzo’s expenditure is EUR 786,166 or +21.34% when the same percentage as the Italian average is applied. In the hypothetical scenario of exclusive biosimilar usage, Abruzzo’s expenditure would be EUR 497,862 or −23.16%.

As Abruzzo is the leading region in this case, projection based on the most efficient region is not valid.

### 3.3. Linear Correlation of Price with Percentage of Use

**Figure 2:**
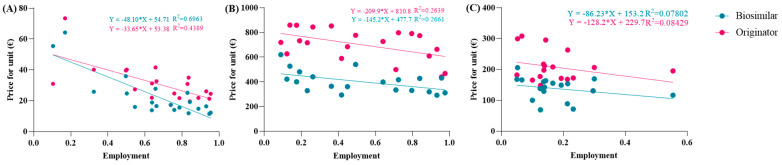
Analysis of the linear correlations between the prices and utilization of (**A**) epoetin, (**B**) etanercept, and (**C**) follitropin.

The spending trend was analyzed by varying the use percentages while keeping the prices of the originator and biosimilar constant, utilizing the prices charged in the Abruzzo region. However, it was hypothesized that as the percentages of biosimilar use increase, their prices might decrease due to the law of supply and demand. Indeed, an observation from the AIFA report indicates that the unit prices charged to individual regions were reduced as the percentages of biosimilar use increased. This led to an investigation into whether this relationship exhibited a linear correlation. To assess this, the price values and percentages of biosimilar utilization rates were compared in a Cartesian plane, along with those of the originator. Figure 2A reveals a strong linear correlation between price and percentage of use, particularly for the biosimilar with an R^2^ value approaching 0.7, which is considered optimal for real data analysis. Comparable graphic correlations were also conducted for etanercept and follitropin. The R^2^ values obtained were lower for etanercept and further decreased for follitropin (Figure 2B,C). Despite this behavior, the correlation is considered significant since it involves real data. Additionally, upon analyzing the price values described by the line, they closely approximate the real regional data.

### 3.4. Adaptive Pricing Function

#### 3.4.1. First Function

**Figure 3:**
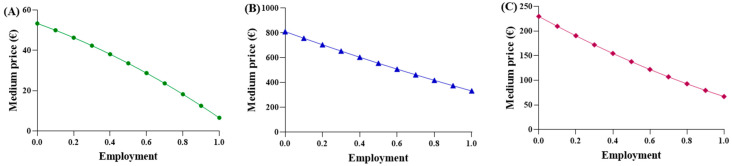
Trends of the first adaptive price function for (**A**) epoetin, (**B**) etanercept, and (**C**) follitropin.

A function was proposed to calculate an “adaptive” average price, considering two key factors: (a) the increasing use of biosimilar drugs, which are priced lower than the originator (modeled linearly); (b) the decrease in unit price resulting from higher procurement volumes of biosimilars, in agreement with the principles of supply and demand. Subsequently, it was recognized that the function should also account for the behavior of the originator. As the percentage of biosimilar utilization increases, the originator utilization proportionally decreases, resulting in higher costs. Function (4) is considered suitable to express and represent an average purchase price in comparison to the biosimilar percentage, using the parabolic function (Figure 3).

The observed average prices ranged from EUR 53.88 to EUR 6.61 for epoetin, from EUR 810.83 to EUR 332.50 for etanercept, and from EUR 229.69 to EUR 67.01 for follitropin, with the two extreme values representing the prices of each drug at its maximum quantity. The average prices derived from the function were evaluated based on the actual percentages in Abruzzo, and the following results were obtained: epoetin—EUR 33.51 (50.4% biosimilars); etanercept—EUR 737.13 (13.8% biosimilars); follitropin—EUR 129.20 (55.4% biosimilars). Based on these results, a convex parabola was observed for epoetin, while for etanercept and follitropin, a concave shape was obtained. In a parabolic function, the concavity or convexity depends on the sign of the coefficient of x^2^, represented by (c-m), as well as on the angular coefficients of the biosimilar and originator lines. If the sum of these coefficients is less than zero, it results in a convex parabola; conversely, if the sum is greater than zero, a concave parabola is generated. Thus, the visual observations are validated: [epoetin: (c-m) = −48.1 + 33.6 = −14.5 < 0 convex; etanercept: (c-m) = −145.2 + 209.9 = 64.7 > 0 concave; follitropin: (c-m) = −86.2 + 128.2 = 42 > 0 concave]. Subsequently, shortcomings in the function and in Figure 1 were identified. Despite comparable utilization rates, there were disparities in the prices observed across the regions. For example, in the case of follitropin, regions with similar biosimilar usage percentages (12–13%) exhibited prices ranging from EUR 69.00 to over EUR 163.00, likely attributed to differences in population. This difference cannot be totally attributed to the different bargaining capabilities among the regions, but rather to their different levels of openness. Assuming a constant incidence of a pathology, as the number of inhabitants of a region increases, the quantity of required drugs will also increase. Subsequently, we decided to relate the price to the quantity of drugs purchased (expressed as the number of packages) instead of to the percentage of utilization, which is only an index of quantity. In this case, the results highlight that, although the R^2^ values remained less than optimal due to the wide variability in prices spanning multiple orders of magnitude, the lines accurately depict reality, except for the originator line in the etanercept graph, which unexpectedly shows a positive slope. This contradicts the previous observations, since this trend indicates a higher drug price for larger purchase quantities.

#### 3.4.2. Second Function

**Figure 4:**
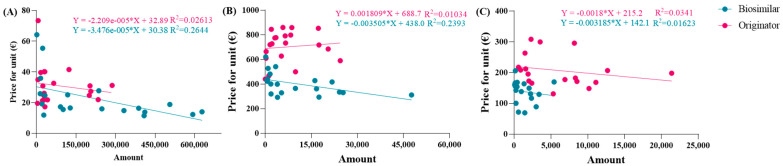
Linear correlations between prices and quantity for (**A**) epoetin, (**B**) etanercept, and (**C**) follitropin.

The same operations were carried out for the second function. To align the trends more closely with reality, the equations of the new dispersion lines obtained from the quantity graphs were used (Figure 4A–C). As a result, the total cost of pharmaceutical spending, in terms of quantity, is represented by the parabolic Function (8). Unfortunately, we did not obtain an average price value from this equation, but instead determined a “total expenditure” as a function of a specific quantity. The average price, denoted by p, was obtained by dividing by the number of packages in Equation (9), which was then simplified into the final Function (10). In this proposed function, the coefficients c, d, m, and n were derived from the national trends and, therefore, remained constant across the regions, while the quantities used were specific to each region.

**Figure 5:**
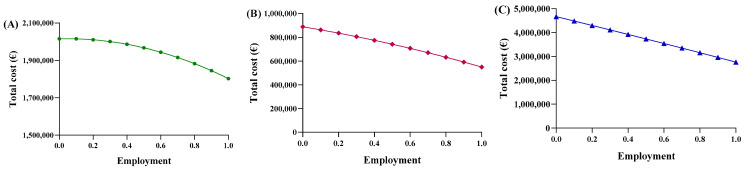
Trends of the second adaptive price function for (**A**) epoetin, (**B**) etanercept, and (**C**) follitropin as a function of the total cost.

**Figure 6:**
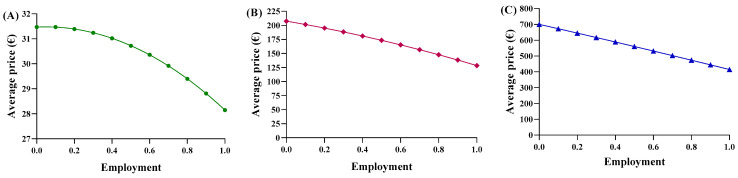
Trends of the second adaptive price function for (**A**) epoetin, (**B**) etanercept, and (**C**) follitropin as a function of the average price.

Consequently, each region yielded a different relationship between the average price and the percentage of utilization. Based on the trends in Abruzzo, epoetin spending ranges from EUR 2,015,400 to EUR 1,802,773, resulting in savings of EUR 201,914 (Figure 5A). The unit price ranges from EUR 31.47 to EUR 28.15 (Figure 6A), with a delta percentage of −10.55%. For etanercept, the spending value ranges from EUR 4,668,454 to EUR 2,762,943, resulting in savings of EUR 1,905,511 (Figure 5B). The unit price ranges from EUR 700.65 to EUR 414.67 (Figure 6B), with a percentage delta of −40.82%.

Finally, follitropin reveals a spending value ranging from EUR 887,768 to EUR 549,425, resulting in savings of EUR 338,343 (Figure 5C). The unit price ranges from EUR 207.51 to EUR 128.43 (Figure 6C), with a percentage delta of −38.12%. Analysis of the coefficients of x^2^ (c-m) yielded a value less than zero for each of the three drugs, indicating a convex parabolic shape for all of them. The average price obtained from the function were analyzed based on the actual Abruzzo percentages, and the following results were obtained: epoetin—EUR 30.72 (50.4% biosimilars); etanercept—EUR 662.54 (13.8% biosimilars); follitropin—EUR 168.98 (55.4% biosimilars). By comparing the real average price with the prices obtained from the functions, it was possible to determine whether the price bargaining capacity of the Abruzzo region is effective. For epoetin, the region is considered to negotiate effectively according to the first function, but not according to the second. For etanercept, both functions indicate a lower price than is applied, indicating that the region does not negotiate effectively. For follitropin, the first function yields a negative response, while the second function yields a positive result.

### 3.5. Regional Ranking Models: The K_pt_

These evaluations suggest the introduction of a coefficient to quantify the judgment, measuring the deviation between real and theoretical prices. This coefficient can rank regions based on their bargaining power. Negative values of the index indicate regions with strong negotiating power, where they pay less than the ideal price trend for the drug. Positive values indicate regions with lower bargaining power, where they pay more than the ideal price trend. The deviation was assessed against the ideal trend from the quantity function, which is considered more realistic. The coefficient represents the percentage deviation from the theoretical price. As shown in Table 4, Table 5 and Table 6, northern regions show more favorable outcomes with negative coefficients, indicating a higher negotiating capacity, while southern regions tend to have positive coefficients, suggesting lower bargaining power. For example, Abruzzo ranks 13th for epoetin, with a low positive index, 20th for etanercept, with a significant positive index, and 8th for follitropin, with a positive index and good value.

### 3.6. Centralized Purchasing Centers

**Figure 7:**
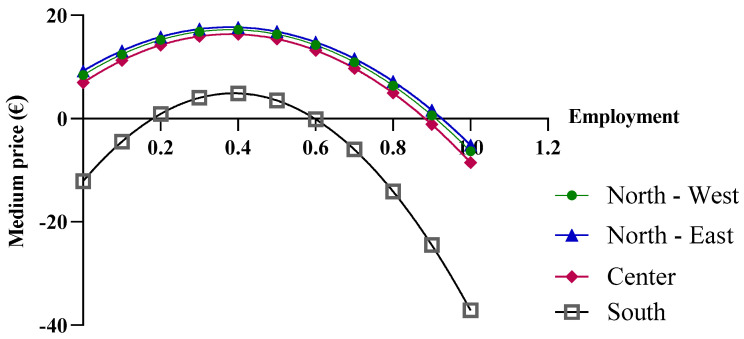
Price trends of epoetin based on centralized purchasing centers.

The final economic analysis was based on the criterion of the price dependence on purchase quantities. Since the pricing criteria for biological drugs recognize the law of supply and demand, with the goal of reducing regional spending, centralized purchasing centers were proposed for “macro-regions”, formed based on the geographical proximity of the regions. More specifically, the macro-regions are divided as follows: North-West (Aosta Valley, Ligury, Piedmont, and Lombardy); North-East (Friuli, Emilia, Veneto, and Trento); Center (Tuscany, Marche, Umbria, Abruzzo, Lazio, and Sardinia); and South (Apulia, Molise, Campania, Sicily, Calabria, and Basilicata). By applying Function (10) and considering the total number of packages purchased by the members of each macro-region, different results were obtained. In the previous analysis, the function used took into account the real purchase quantities, for which the assumption of a linear variation in the prices of drugs was acceptable. However, in the case of the macro-regions, the cumulative quantities significantly exceed the regional quantities; consequently, the linearity assumption is no longer valid (Figure 7).

**Figure 8:**
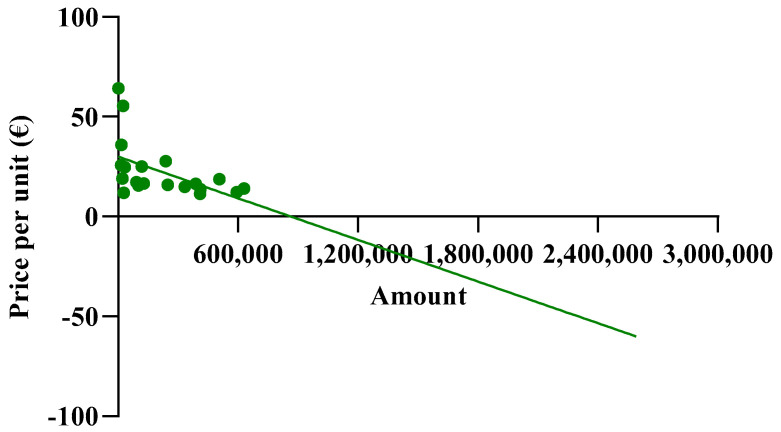
Extension of the biosimilar curve reported in Figure 4.

As shown in Figure 8, when more than one million biosimilar packages are purchased, the price becomes negative. The price predictions obtained from the function indicate an increase in purchase prices with the initial growth in the biosimilar percentages, up to approximately 40%, while negative purchase prices appear at percentages that approach 100%. For the North-East, North-West, and Center macro-regions, the results obtained from the function are reliable when the biosimilar utilization rates ranges from 40% to 70%, while for the Southern macro-region, the results are unrealistic. It should be stated that the function is useful when the biosimilar percentage rate of use is 40%, at which point a price of EUR 4.89 is obtained. This price is below the cost price and, therefore, the forecast is not reliable.

**Figure 9:**
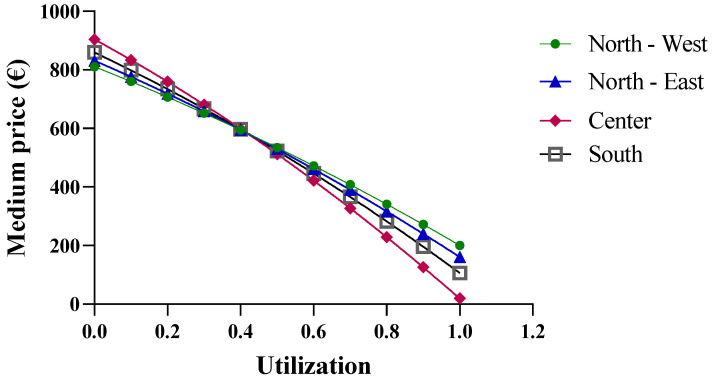
Price trends of etanercept based on centralized purchasing centers.

In the case of etanercept, the results obtained from the function are valid (Figure 9). Only results reported for a biosimilar utilization rate close to 100% could be excluded, as they are outside the valid range. An interesting observation is that at low biosimilar utilization rates, the macro-regions with greater purchase quantities show higher prices. This is primarily due to the price/quantity curve of the originator (Figure 4B), which has a positive slope. This trend implies an equal average price across all macro-regions with different purchase quantities, which occurs at a biosimilar utilization rate of about 40%.

**Figure 10:**
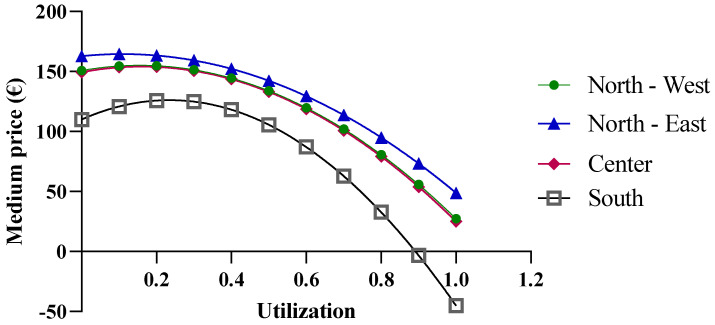
Price trends of follitropin based on centralized purchasing centers.

The results disagree with the expectations and, consequently, highlight a limitation of the developed model and the database used. Questionable data may be attributed to flaws in the data collection phase. Alternatively, it is possible that the regions have not carried out effective negotiations for this drug. In the case of follitropin, as the quantities increase, the price shows a less realistic trend. For the Southern macro-region, which has the highest utilization rate, the price increases by up to 30%, but it becomes negative starting at a utilization rate of 80% (Figure 10). In contrast, negative values no longer appear for the lowest purchasing quantities in the North-East, with the price only increasing up to a utilization rate of 20%.

## 4. Conclusions

Healthcare technologies are becoming increasingly complex while resources remain limited, necessitating a focus on economic sustainability. HTA serves as vital support in this regard. Biosimilars, as an additional therapeutic option, offer significant cost savings, reaching up to 40% through increased utilization. Further savings are anticipated with the implementation of centralized purchasing centers, thus minimizing unit prices. This work addressed the challenge of determining the right price for biosimilar drugs through highlighting the issue of sustainability, understood as recognizing the cost of technology based on the expected value for the population. Policies that are too extreme and aimed only at saving money could lead to distrust in the companies producing biosimilar drugs, thus hindering competition. Furthermore, as is already happening, there could be drugs with expired patent coverage that do not have related biosimilars on the market, precisely because the market is not considered attractive. This phenomenon can lead to a decrease in the diversity of pharmacological offerings, to the detriment of both patients and the healthcare system. These projections rely on mathematical functions aimed at aiding healthcare decisionmakers, rather than guiding pharmaceutical pricing activities. Expanding beyond linear functions to include exponential, logarithmic, and other complex models may better represent real trends and widen the applicability of this analysis. Moreover, updating data beyond 2017 to include newer biosimilar introductions, such as heparins, teriparatide, and trastuzumab, is essential. Disseminating scientific information to encourage biosimilar usage is crucial to prevent their perception as solely a cost-saving strategy. For more reliable results, future surveys should target professionals actively prescribing biological drugs. HTA is a potent tool for healthcare system modernization, with biosimilar technology facilitating the rapid release of resources, as seen in EU countries with automatic substitutability. Achieving the core objectives of HTA benefits not only healthcare, but also the broader social system.

## Figures and Tables

**Table 1:** Data for epoetin.

Region	Population	Medium Price	Total Amount	*Per* *Capita* Spending	Biosimilar (%)	Biosimilar Price	Biosimilar Amount	Originator (%)	Originator Price	Originator Amount
**EMILIA-ROMAGNA**	4,448,841	12.88	621,732	1.80	95.4	12.32	593,133	4.6	24.49	28,600
**PIEDMONT**	4,392,526	12.01	431,572	1.18	94.7	11.49	408,699	5.3	21.30	22,873
**TUSCANY**	3,742,437	17.04	417,291	1.90	93.0	16.36	388,080	7.0	26.07	29,210
**VENETO**	4,907,529	15.63	373,638	1.19	88.8	14.85	331,790	11.2	21.81	41,847
**BOLZANO**	524,256	19.21	25,380	0.93	84.2	19.14	21,370	15.8	19.58	4010
**TRENTO**	538,604	15.73	33,213	0.97	83.5	11.93	27,733	16.5	34.96	5480
**LIGURIA**	1,565,307	26.12	142,628	2.38	82.9	25.13	118,238	17.1	30.92	24,389
**FRIULI-VENEZIA GIULIA**	1,217,872	16.90	128,994	1.79	78.6	15.59	101,389	21.4	21.71	27,605
**SICILY**	5,056,641	16.59	829,057	2.72	75.8	14.01	628,426	24.2	24.67	200,632
**SARDINIA**	1,653,135	17.29	123,340	1.29	73.9	17.29	91,148	26.1	17.29	32,192
**MARCHE**	1,538,055	22.00	193,655	2.77	66.2	16.57	128,200	33.8	32.64	65,455
**APULIA**	4,063,888	32.48	360,345	2.88	65.7	27.75	236,746	34.3	41.54	123,598
**CAMPANIA**	5,839,084	23.33	793,395	3.17	63.8	18.83	506,186	36.2	31.26	287,209
**LOMBARDY**	10,019,166	16.76	645,626	1.08	63.7	13.80	411,264	36.3	21.95	234,362
**LAZIO**	5,898,124	21.15	454,560	1.63	54.6	15.98	248,190	45.4	27.37	206,370
**ABRUZZO**	1,322,247	32.42	64,032	1.57	50.2	24.71	32,144	49.8	40.19	31,888
**BASILICATA**	570,365	37.77	31,712	2.10	49.7	35.91	15,761	50.3	39.61	15,951
**UMBRIA**	888,908	35.52	43,294	1.73	32.4	25.81	14,027	67.6	40.17	29,267
**MOLISE**	310,449	71.87	7948	1.84	16.9	64.20	1343	83.1	73.43	6605
**CALABRIA**	1,965,128	33.52	226,881	3.87	10.4	55.43	23,596	89.6	30.98	203,285
**ITALY**	**60,589,445**	**19.62**	**5,867,479**	**1.90**	**71.2**	**16.59**	**4,177,645**	**28.8**	**27.11**	**1,689,834**
**AOSTA VALLEY**	**126,883**	**5.26**	**4101**	**0.17**	**97.0**	**5.26**	**3978**	**3.0**	**5.26**	**123**

**Table 2:** Data for etanercept.

Region	Population	Medium Price	Total Amount	*Per* *Capita* Spending	Biosimilar (%)	Biosimilar Price	Biosimilar Amount	Originator (%)	Originator Price	Originator Amount
**TUSCANY**	3,742,437	315.23	48,794	4.11	97.6	311.47	47,623	2.4	468.14	1171
**AOSTA VALLEY**	126,883	431.86	529	1.80	95.7	431.47	506	4.3	440.54	23
**BOLZANO**	524,256	317.60	4292	2.60	93.2	292.37	4000	6.8	663.40	292
**TRENTO**	538,604	351.74	2128	1.39	89.0	319.91	1894	11.0	609.27	234
**PIEDMONT**	4,392,526	483.40	19,264	2.12	83.9	427.60	16,162	16.1	774.18	3101
**EMILIA-ROMAGNA**	4,448,841	424.69	31,846	3.04	79.6	330.73	25,349	20.4	791.32	6496
**SICILY**	5,056,641	521.93	30,034	3.10	72.4	416.86	21,745	27.6	797.55	8289
**VENETO**	4,907,529	382.46	34,003	2.65	71.0	334.47	24,142	29.0	499.95	9861
**LIGURIA**	1,565,307	516.59	6121	2.02	64.1	398.91	3923	35.9	726.71	2197
**FRIULI-VENEZIA GIULIA**	1,217,872	660.80	6874	3.73	49.3	541.35	3389	50.7	776.95	3485
**LAZIO**	5,898,124	539.49	37,171	3.40	44.9	361.42	16,690	55.1	684.60	20,481
**LOMBARDY**	10,019,166	466.22	41,906	1.95	41.7	293.43	17,475	58.3	589.81	24,431
**CAMPANIA**	5,839,084	676.35	27,108	3.14	36.2	364.70	9813	63.8	853.18	17,295
**BASILICATA**	570,365	738.91	2671	3.46	26.2	441.04	700	73.8	844.66	1971
**APULIA**	4,063,888	626.92	22,753	3.51	23.2	328.38	5279	76.8	717.10	17,474
**MARCHE**	1,538,055	684.74	8176	3.64	19.0	481.03	1553	81.0	732.52	6623
**CALABRIA**	1,965,128	779.40	10,464	4.15	17.4	400.83	1821	82.6	859.15	8643
**ABRUZZO**	1,322,247	813.67	6663	4.10	13.8	527.03	919	86.2	859.56	5743
**UMBRIA**	888,908	601.84	5997	4.06	12.2	422.22	732	87.8	626.80	5265
**MOLISE**	310.449	710.55	1700	3.89	8.9	620.22	151	91.1	719.37	1548
**ITALY**	**60,589,445**	**529.48**	**345,585**	**3.02**	**56.1**	**377.50**	**193,873**	**43.9**	**723.70**	**151,712**
**SARDINIA**	**1,653,135**	**612.57**	**12,846**	**4.76**	**92.2**	**615.90**	**11,844**	**7.8**	**573.21**	**1002**

**Table 3:** Data for follitropin.

Region	Population	Medium Price	Total Amount	*Per* *Capita* Spending	Biosimilar (%)	Biosimilar Price	Biosimilar Amount	Originator (%)	Originator Price	Originator Amount
**ABRUZZO**	1,322,247	151.44	4278	0.49	55.4	116.37	2370	44.6	195.00	1908
**SICILY**	5,056,641	195.38	18,117	0.70	30.0	169.46	5435	70.0	206.49	12,682
**SARDINIA**	1,653,135	130.97	7573	0.60	29.6	130.97	2242	70.4	130.97	5332
**LIGURY**	1,565,307	148.95	2627	0.25	23.2	71.47	610	76.8	172.36	2018
**BASILICATA**	570,365	239.64	1904	0.80	21.4	153.81	407	78.6	263.01	1497
**EMILIA**	4,448,841	151.37	14,107	0.48	21.3	88.90	3005	78.7	168.28	11,103
**VENETO**	4,907,529	167.02	10,578	0.36	19.3	149.21	2042	80.7	171.28	8536
**BOLZANO**	524,256	199.42	1078	0.41	16.6	155.55	179	83.4	208.15	899
**APULIA**	4,063,888	276.63	9549	0.65	14.3	163.61	1366	85.7	295.49	8183
**CAMPANIA**	5,839,084	188.53	24,777	0.80	13.7	129.25	3394	86.3	197.94	21,383
**TRENTO**	538,604	209.56	694	0.27	13.6	160.20	94	86.4	217.33	600
**TUSCANY**	3,742,437	138.21	11,643	0.43	12.6	69.22	1467	87.4	148.16	10,176
**PIEDMONT**	4,392,526	172.40	7898	0.31	12.5	138.21	987	87.5	177.28	6911
**FRIULI VENEZIA GIULIA**	1,217,872	159.10	2679	0.35	10.0	100.05	268	90.0	165.66	2411
**MARCHE**	1,538,055	298.69	2472	0.48	6.6	166.10	163	93.4	308.06	2309
**CALABRIA**	1,965,128	294.56	3736	0.56	5.2	205.72	194	94.8	299.43	3542
**LAZIO**	5,898,124	181.57	8771	0.27	5.0	168.25	439	95.0	182.27	8332
**UMBRIA**	888,908	202.11	1847	0.42	13.9	142.43	257	86.1	211.74	1590
**AOSTA VALLEY**	**126,883**	**172.58**	**118**	**0.16**	**4.3**	**93.22**	**5**	**95.7**	**176.15**	**113**
**LOMBARDY**	**10,019,166**	**310.07**	**25,204**	**0.78**	**4.1**	**0.00**	**1033**	**95.9**	**323.33**	**24,170**
**ITALY**	**60,589,445**	**209.09**	**153,582**	**0.53**	**14.3**	**127.40**	**21,962**	**85.7**	**222.72**	**131,620**
**MOLISE**	**310,449**	**282.02**	**396**	**0.36**	**9.0**	**53.16**	**36**	**91.0**	**304.65**	**361**

**Table 4:** Ranking of regions for epoetin based on K_pt_.

n°.	Region	% Biosimilar	Amount	Real Price	Ideal Price	K_pt_
**1**	**TRENTO**	83.5	33,213	15.73	30.08	−0.48
**2**	**FRIULI**	78.6	128,994	16.90	28.40	−0.41
**3**	**SARDINIA**	73.9	123,340	17.29	28.84	−0.40
**4**	**BOLZANO**	84.2	25,380	19.21	30.22	−0.36
**5**	**PIEDMONT**	94.7	431,572	12.01	18.87	−0.36
**6**	**VENETO**	88.8	373,638	15.63	21.72	−0.28
**7**	**LOMBARDY**	63.7	645,626	16.76	21.73	−0.23
**8**	**MARCHES**	66.2	193,655	22.00	28.24	−0.22
**9**	**LAZIO**	54.6	454,560	21.15	25.58	−0.17
**10**	**TUSCANY**	93.	417,291	17.04	19.68	−0.13
**11**	**LIGURY**	82.9	142,628	26.12	27.78	−0.06
**12**	**EMILIA**	95.4	621,732	12.88	13.49	−0.05
**13**	**ABRUZZO**	50.2	64,032	32.42	30.83	0.05
**14**	**SICILY**	75.8	829,057	16.59	15.72	0.06
**15**	**UMBRIA**	32.4	43,294	35.52	31.54	0.13
**16**	**CALABRIA**	10.4	226,881	33.52	28.91	0.16
**17**	**CAMPANIA**	63.8	793,395	23.33	19.52	0.20
**18**	**BASILICATA**	49.7	31,712	37.77	31.24	0.21
**19**	**APULIA**	65.7	360,345	32.48	25.72	0.26
**20**	**MOLISE**	16.9	7948	71.87	32.35	1.22

**Table 5:** Ranking of regions for etanercept based on K_pt_.

n°	Region	% Biosimilar	Amount	Real Price	Ideal Price	K_pt_
**1**	**BOLZANO**	93.2	4292	317.60	442.02	−0.28
**2**	**TRENTO**	89.0	2128	351.74	459.71	−0.23
**3**	**LOMBARDY**	41.7	41,906	466.22	584.26	−0.20
**4**	**VENETO**	71.0	34,003	382.46	455.84	−0.16
**5**	**UMBRIA**	12.2	5997	601.84	666.09	−0.10
**6**	**LAZIO**	44.9	37,171	539.49	570.19	−0.05
**7**	**APULIA**	23.2	22,753	626.92	650.37	−0.04
**8**	**AOSTA V.**	95.7	529	431.86	447.08	−0.03
**9**	**LIGURIA**	64.1	6121	516.59	520.60	−0.01
**10**	**EMILIA**	79.6	31,846	424.69	420.89	0.01
**11**	**MARCHE**	19.0	8176	684.74	649.66	0.05
**12**	**MOLISE**	8.9	1700	710.55	668.84	0.06
**13**	**TUSCANY**	97.6	48,794	315.23	281.38	0.12
**14**	**PIEDMONT**	83.9	19,264	483.40	431.79	0.12
**15**	**CAMPANIA**	36.2	27,108	676.35	605.35	0.12
**16**	**SICILY**	72.4	30,034	521.93	456.19	0.14
**17**	**FRIULI**	49.3	6874	660.80	562.41	0.17
**18**	**BASILICATA**	26.2	2671	738.91	624.96	0.18
**19**	**CALABRIA**	17.4	10,464	779.40	656.78	0.19
**20**	**ABRUZZO**	13.8	6663	813.67	662.53	0.23

**Table 6:** Ranking of regions for follitropin based on K_pt_.

n°	Region	% Biosimilar	Amount	Real Price	Ideal Price	K_pt_
**1**	**SARDINIA**	29.6	7573	130.97	184.70	−0.29
**2**	**TUSCANY**	12.6	11,643	138.21	189.41	−0.27
**3**	**LIGURY**	23.2	2627	148.95	195.02	−0.24
**4**	**FRIULI**	10.0	2679	159.10	203.92	−0.22
**5**	**EMILIA**	21.3	14,107	151.37	181.87	−0.17
**6**	**PIEDMONT**	12.5	7898	172.40	194.80	−0.12
**7**	**VENETO**	19.3	10,578	167.02	187.45	−0.11
**8**	**ABRUZZO**	55.4	4278	151.44	168.99	−0.10
**9**	**LAZIO**	05.0	8771	181.57	197.25	−0.08
**10**	**BOLZANO**	16.6	1078	199.42	201.64	−0.01
**11**	**UMBRIA**	13.9	1847	202.11	202.48	0.00
**12**	**TRENTO**	13.6	694	209.56	204.30	0.03
**13**	**CAMPANIA**	13.7	24,777	188.53	170.50	0.11
**14**	**SICILY**	30.0	18,117	195.38	172.09	0.14
**15**	**BASILICATA**	21.4	1904	239.64	197.18	0.22
**16**	**CALABRIA**	5.2	3736	294.56	205.34	0.43
**17**	**APULIA**	14.3	9549	276.63	191.52	0.44
**18**	**MARCHE**	6.6	2472	298.69	206.48	0.45

## Data Availability

The original contributions presented in this study are included in the article. Further inquiries can be directed to the corresponding author.
